# Pyrite Passivation by Triethylenetetramine: An Electrochemical Study

**DOI:** 10.1155/2013/387124

**Published:** 2013-01-28

**Authors:** Yun Liu, Zhi Dang, Yin Xu, Tianyuan Xu

**Affiliations:** ^1^Department of Environmental Science and Engineering, Xiangtan University, Xiangtan 411105, China; ^2^The Key Lab of Pollution Control and Ecosystem Restoration in Industry Clusters, Ministry of Education, Guangzhou 510006, China; ^3^Higher Education Mega Center, School of Environmental Science and Engineering, South China University of Technology, Guangzhou 510006, China

## Abstract

The potential of triethylenetetramine (TETA) to inhibit the oxidation of pyrite in H_2_SO_4_ solution had been investigated by using the open-circuit potential (OCP), cyclic voltammetry (CV), potentiodynamic polarization, and electrochemical impedance (EIS), respectively. Experimental results indicate that TETA is an efficient coating agent in preventing the oxidation of pyrite and that the inhibition efficiency is more pronounced with the increase of TETA. The data from potentiodynamic polarization show that the inhibition efficiency (*η*%) increases from 42.08% to 80.98% with the concentration of TETA increasing from 1% to 5%. These results are consistent with the measurement of EIS (43.09% to 82.55%). The information obtained from potentiodynamic polarization also displays that the TETA is a kind of mixed type inhibitor.

## 1. Introduction

Pyrite, FeS_2_, is one of the most common sulfide minerals. It is frequently present in tailings, waste rock dumps, many valuable mineral raw materials, and coal [1]. It is easy to be oxidized under natural weathering conditions. The oxidation of pyrite results in sulfuric acid and toxic trace metals formation in acid mine drainage (AMD), which is one of the most serious environmental problems facing the mining industry [2]. That is why many studies have been carried out on the mechanism of pyrite's oxidation during the last six decades by investigators from different areas, such as metallurgy and environment science [3–9]. Now researchers have found that the oxygen and ferric iron play a very important role for the pyrite's oxidation [10, 11] and that some acidophilic microorganisms, for example, *Acidithiobacillus ferrooxidans* [12] and *Leptospirillum ferrooxidans* [13], can accelerate the oxidation of pyrite greatly. According to the knowledge of people for the mechanism of pyrite decomposition, if it is no contact between pyrite and oxidants (e.g., O_2_ and Fe^3+^), the rate of pyrite oxidation could be suppressed. For years, several techniques have been developed to reduce the oxidation of sulfide minerals, including bactericides [14], neutralization [15, 16], and cover treatment [17–19]. However, most of these technologies are costly, short-term solutions, and difficult to apply.

In parallel, many researchers have used certain chemical reagents that can create effective oxygen barriers to protect the surface of iron sulfide from oxidation. For example, both iron phosphate precipitates and silica precipitates have been shown to suppress pyrite oxidation efficiently. However, these treatments require initial surface oxidation with hydrogen peroxide, which is difficult to handle in a real application [20, 21]. Similarly, although some passivating agents such as acetyl acetone, humic acids, ammonium lignosulfonates, oxalic acid, and sodium silicate also have the capability to inhibit pyrite from oxidation, these treatments also need peroxidation, and the coating with oxalic acid requires a temperature control at 65°C [22]. In addition, Elsetinow et al. [23] have concluded that the formation of a passivating layer on the pyrite surface after exposure to the lipid could suppress pyrite oxidation by either interrupting the advection of aqueous oxidants or the electron transfer between oxidants and the pyrite. Using the property of formation of strong insoluble chelating complex with Fe^3+^, Lan et al. [24] have investigated the possibility of using 8-hydroxyquinoline as a passivating agent, and they demonstrated that the oxidation rates of pyrite could be reduced remarkably. In recent years, our laboratory has also developed a new passivating agent: triethylenetetramine (TETA) [25]. Compared to the coating agents mentioned above, TETA is currently used in the floatation process of sulfide minerals in Inco Limited as depressant; therefore, it does not represent any extra cost. On the other hand, TETA is a base which can neutralize protons produced in the oxidation of the sulphide minerals. TETA has already been proved that it could retard the oxidation of pyrrhotite and need not initial oxidation [25, 26]. However, there is not date on the capability of TETA to passivate pyrite. In addition, all the studies cited above were carried out by the method of extraction using hydrogen peroxide or atmospheric oxygen as oxidant to test the coating effectiveness of different passivating agents. These processes usually require long times, and, moreover, the operation is complicated as the quantity of dissolved metal ions need to be monitored by techniques such as spectrophotometer [23].

As the simplicity, efficacy, and low cost of these methods, electrochemical techniques are used extensively to investigate the corrosion of steel [27–30]. Nowadays, electrochemical techniques have been becoming essential measurements to evaluate the effect of inhibitors on the corrosion inhibition of steel. Although pyrite is not a very good electrical conductor, its oxidation is usually described in terms of electrochemical corrosion mechanisms developed for metals [31, 32]. Therefore, electrochemical methods can be chosen to study the corrosion inhibition behavior of passivating agents on pyrite.

The main aim of this study is to test the coating effectiveness of triethylenetetramine (TETA) on pyrite using the open-circuit potential (OCP), cyclic voltammetry (CV), potentiodynamic polarization, and electrochemical impedance spectroscopy (EIS).

## 2. Experimental Methods

### 2.1. Mineral Samples Preparation

**Figure 1:**
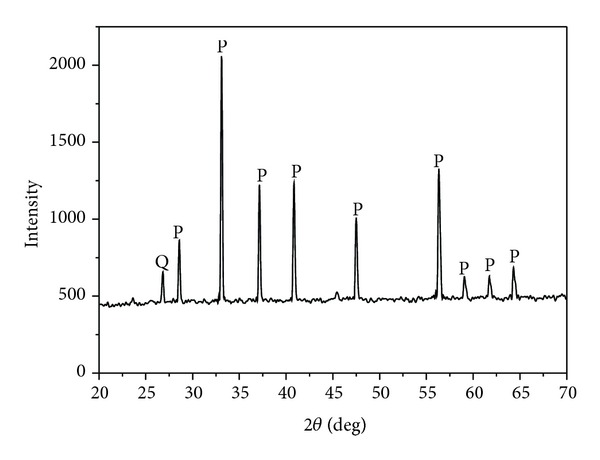
XRD spectra of the pyrite. P: Pyrite, Q: quartz.

Natural pyrite was obtained from the Dabaoshan sulfur-polymetallic mines in the north of Guangdong Province, China. Its chemical composition analysis by X-ray fluorescence (XRF) is listed in Table 1. The XRD pattern (Figure 1) of the crushed sample is typical that was expected for pyrite and showed that it was including trace of quartz. The material was ground with an agate mortar and then sieved to isolate particles with a diameter of less than 75 *μ*m and stored in a vacuum desiccator before usage.

The pyrite sample was submitted to passivation by various concentrations of TETA solution. 1 g of the pyrite powder was precisely weighed in a 50 mL glass beaker, and then 1 mL of coating solution was added. Samples were rinsed well with the coating solution and dried overnight in a vacuum desiccator. All of these reagents in this experiment were analytical grade. Milli-Q water was used to prepare all the solutions. After the coating step, the particles were used to construct carbon paste electrodes.

These electrodes were consisted of 1.0 g graphite, 0.4 mL paraffin oil, and 0.5 g pristine or coated pyrite. The method of the construction of C paste electrode was described by Arce and González [33], A total of 1.0 g of graphite was pulverized together with 0.5 g of pristine or coated pyrite in an agate mortar, then 0.4 mL of silicon oil was added in the powder and mixed to obtain a homogeneous paste. This paste was placed in a 7 cm long and 0.5 cm diameter glass tube. The electrode surface was compacted on a plate glass to make it flat, and its apparent active area was around 0.196 cm^−2^. From the other end of the tube, a copper wire with diameter of 1.5 mm was immersed in the paste as the conductor.

Prior to the electrochemical study, the surface of these C paste electrodes was sequentially polished with 300, 600, and 1200 grade silicon carbide paper. And then these electrodes were rinsed with distilled water and quickly transferred to the cell.

### 2.2. Electrochemical Analysis

The electrochemical measurements were performed in a typical electrochemical cell (200 mL) with three electrodes: the working electrode (Carbon paste electrode with pristine or coated pyrite), the counter electrode (a platinum foil electrode with 1 cm^2^ area), and the reference electrode (KCl-saturated calomel electrode). The electrolyte was 0.5 mol L^−1^ H_2_SO_4_ solution.

The electrochemical measurements were performed by an electrochemical workstation (2273, Parstart), and the experimental data was recorded on a personal computer with suitable software. Cyclic voltammetry (CV) experiments were conducted, starting from open-circuit potentials (OCPs), at a sweep rate of 100 mV s^−1^, and the scan range was from −0.6 V/SCE to +0.8 V/SCE. Polarization curves were measured over the range of OCP ±200 mV at a constant rate of potential change of 1 mV s^−1^. From these polarization curves, corrosion current densities (*j*_corr_) of different pyrite electrodes were obtained. Then, the inhibition efficiency of TETA on pyrite can be calculated by using the following equation [27]:

(1a) $$\begin{array}{c} \eta \left(\%\right)=\frac{j_{\text{corr}}-j_{\text{corr}(\text{inh})}}{j_{\text{corr}}}, \end{array}$$

where *j*_corr_ and *j*_corr(inh)_ were the corrosion current density of pristine and coated pyrite samples, respectively.

**Figure 2:**
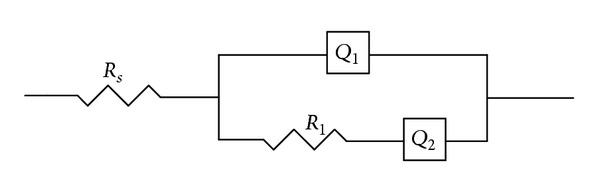
Equivalent electrical circuits proposed for fitting impedance spectra of pyrite oxidation.

The impedance spectra were obtained by applying a signal on the OCP with a frequency range from 5 × 10^5^ to 1 × 10^−2 ^Hz with a sinusoidal excitation signal of 10 mV. The impedance data were analyzed using ZSimpWin software. The equivalent circuit *R*_*s*_(*Q*_1_(*R*_1_ *Q*_2_)), as shown in Figure 2, was used to fit these impedance data. As Bevilaqua et al. [34] suggested, this equivalent circuit was simplified from the circuit of *R*_*s*_(*R*_1_ *Q*_2_(*R*_2_ *Q*_2_)), and it described a response of the corrosion process occurring at the open-circuit potential due to parallel anodic and cathodic reactions. In the circuit of *R*_*s*_(*Q*_1_(*R*_1_ *Q*_2_)), *R*_*s*_ represented the solution resistance, *R*_1_ was the charge transfer resistance in the initial stage of pyrite oxidation, *Q*_1_ was the constant phase element which was associated with the capacitor of the double layer of the electrode/electrolyte interface with passive film, and *Q*_2_ represented the diffusion impedance component, a reaction limited by the diffusion of oxygen. According to the values of charge transfer resistance, the inhibition efficiency (IE) was obtained by using the following equation [27]:

(1b) $$\begin{array}{c} \text{IE}=\frac{R_{1}^{-1}-{R_{1(\text{inh})}}^{-1}}{R_{1}^{-1}}, \end{array}$$

where *R*_1_ and *R*_1(inh)_ were the charge transfer resistance values of pristine and coated pyrite samples, respectively.

All of the above measurements were carried out in static conditions. All potentials quoted in this paper are referenced to the saturated calomel electrode (SCE).

## 3. Results and Discussion

### 3.1. Open-Circuit Potential Measurements

**Figure 3:**
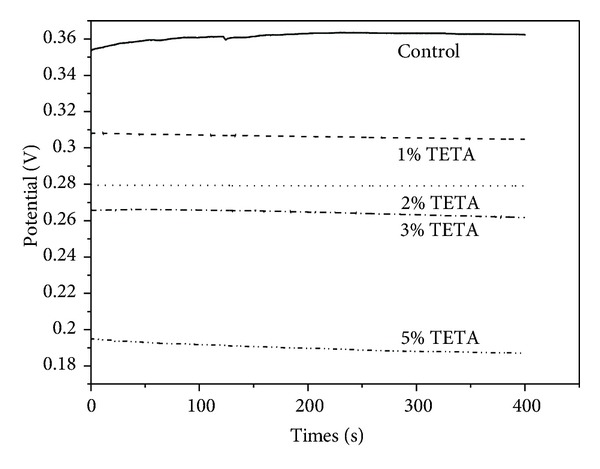
Open-circuit potentials of the pyrite electrodes coated by different concentration of TETA.

Figure 3 shows the OCPs of the pyrite electrodes coated by different concentrations of TETA. The OCP of the uncoated sample (denoted as control) was 362.8 mV, and the OCPs of pyrite samples coated by 1% TETA, 2% TETA, 3% TETA, and 5% TETA were 304.8 mV, 279.2 mV, 261.7 mV, and 187.0 mV, respectively. It is obvious that the OCPs of coated samples were lower than that of uncoated pyrite. This phenomenon was ascribed to the relatively low redox potential of TETA [26].

### 3.2. Cyclic Voltammetry Measurements

**Figure 4:**
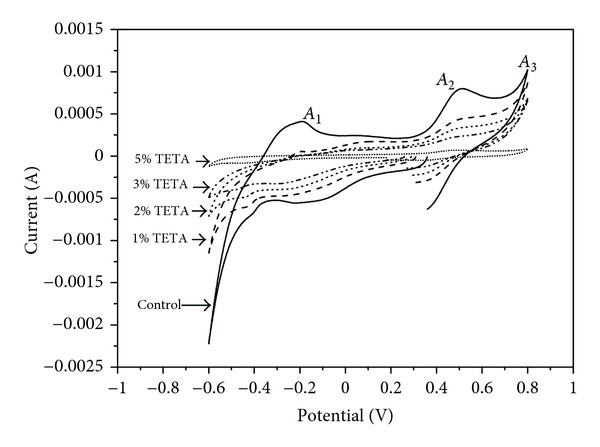
Cyclic voltammograms of pyrite electrodes coated by different concentration of TETA, initiated in the negative direction.

The CV curves of the pristine and coated pyrite electrodes in 0.5 mol L^−1^ H_2_SO_4_ solution obtained by sweeping the potential from OCP towards negative direction are shown in Figure 4. The shape of the voltammetric curve was not significantly influenced by the presence of TETA, indicating that the inhibitor does not change the mechanism of pyrite oxidation. These curves were similar to the other reported results [35, 36], in which the reduction peaks between −0.4 V and −0.2 V can be interpreted as two possible reactions: (1) the reduction of S formed during the handling and preparation of samples and (2) the reduction of FeS_2_(s) to form FeS(s) and H_2_S. The reversal of potential scan produces three anodic current peaks: A_1_, A_2_, and A_3_. A_1_ is attributed to the oxidation of the H_2_S formed electrochemically during the oxidation scan. A_2_ results from the oxidation of pyrite, via two steps [37, 38].

The first step is

(2) $$\begin{array}{c} \text{Fe}{\text{S}}_{2}\to \text{F}{\text{e}}^{2+}+2{\text{S}}^{0}+2{\text{e}}^{-} \end{array}$$

The second step is:

(3) $$\begin{array}{c} \text{F}{\text{e}}^{2+}+3{\text{H}}_{2}\text{O}\to \text{Fe}{\left(\text{OH}\right)}_{3}+3{\text{H}}^{+}+{\text{e}}^{-} \end{array}$$

At high potentials, the oxidation of sulfur to sulfate is expected to occur [39], contributing to the appearance of the anodic current peak A_3_.

**Figure 5:**
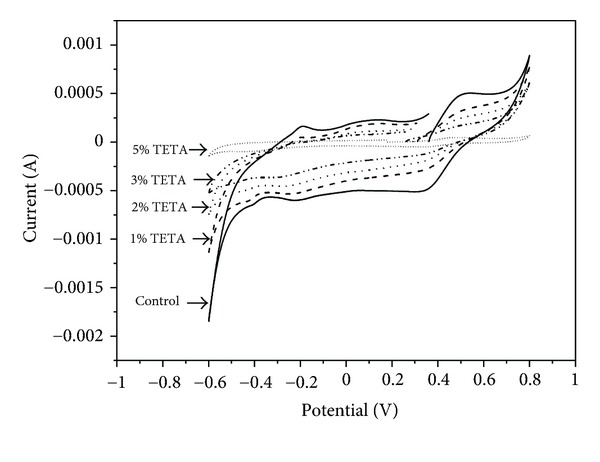
Cyclic voltammograms of pyrite electrodes coated by different concentration of TETA, initiated in the positive direction.

Figure 5 shows the CV curves of the pristine and coated pyrite electrode when the potentials were initially swept from OCP towards the positive direction. In addition to the anodic and cathodic current peaks mentioned above, another cathodic current peak between 0.3 V and 0.4 V appeared when the potential scan was reversed. This peak was attributed to iron oxide reduction.

The evidence of pyrite passivation by TETA can be made by measuring its electrochemical activity [40]. Comparing the CV curves of the pyrite samples coated by various concentrations of TETA, all of the anodic and cathodic current peaks are decreased with an increase in TETA. When 5% of TETA was adopted in the coating treatment, the anodic and cathodic current peaks almost could not be detected. This proves that less-electrochemical activity takes place on the surface of pyrite after being coated by TETA. The decrease of electrochemical activity should be attributed to the formation of a protective layer of TETA on the surface of pyrite samples. As we know, there are several amine molecules in the structure of TETA, consequently, TETA can be absorbed on the pyrite surface through coordination bond formation between the iron in pyrite and to the electron pair on the nitrogen atom [41]. The inhibition efficiencies therefore depend on the coverage area of the adsorbed molecule. With an increase of the concentration of TETA, there is much larger surface area of pyrite coated by TETA, so the inhibition efficiency increases.

### 3.3. Potentiodynamic Polarization Test

**Figure 6:**
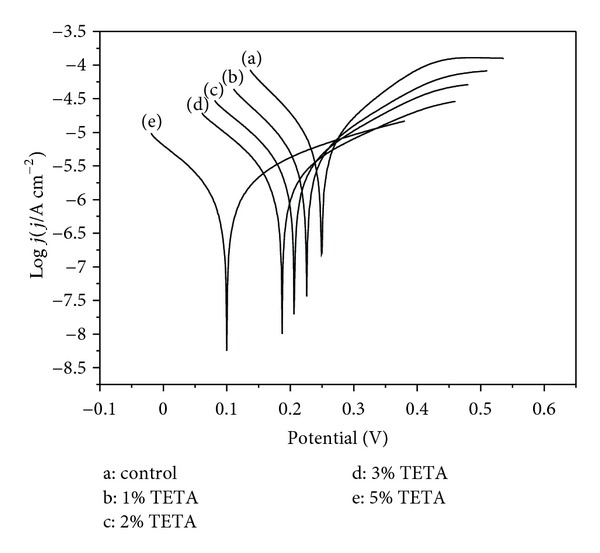
Polarization curves of the pyrite electrodes coated by different concentration of TETA.

The Tafel polarization curves for the pristine and coated pyrite electrodes in 0.5 mol L^−1^ H_2_SO_4_ solution are shown in Figure 6. It was clear that the addition of TETA caused more negative shift in corrosion potential (*E*_corr_) especially in high concentrations. It was consistent with the tendency of OPCs shown in Figure 3. It should be pointed out that the value of the corrosion potential of the same electrode in the same electrolyte was different from the value of the open-circuit potential. This phenomenon was due to the concentrations of reductive products at the interface of the electrode being higher than in a real solution when the applied potential was swept from negative to positive potentials, so the corrosion potential obtained by the polarization curve is lower than the open-circuit potential [42].

From the Tafel polarization curves, some electrochemical corrosion kinetic parameters can be obtained, such as the corrosion potential (*E*_corr_), cathodic and anodic Tafel slopes (*β*_c_, *β*_a_), and corrosion current density (*j*_corr_), which are listed in Table 2.

From the values of cathodic and anodic Tafel slopes, both cathodic and anodic processes were found that are inhibited by the coating of TETA. The cathodic Tafel slopes were slightly decreased from 9.59 dec/V to 7.70 dec/V when the concentration of TETA increasing from 0% to 5%. This indicated that the cathodic reaction (the reduction of FeS_2_) is slightly inhibited by TETA. When the pyrite samples were coated by TETA, the anodic slopes decreased remarkably, which indicates that the inhibition of pyrite dissolution by TETA is mainly controlled by the anodic process. Therefore, TETA can be classified as inhibitors of relatively mixed effect (anodic/cathodic inhibition) in acid solution.

The inhibition efficiencies (*η*%) have been calculated by using (1a), which shows that the inhibition efficiency increases and the corrosion current density decreases with the increase of the TETA concentration. An increase from 42.08% to 80.98% was found when the concentration of TETA increased from 1% to 5%. This could be explained that there is a larger surface area of pyrite sample coated by TETA with the increase of TETA concentration.

### 3.4. Electrochemical Impedance Spectroscopy

**Figure 7:**
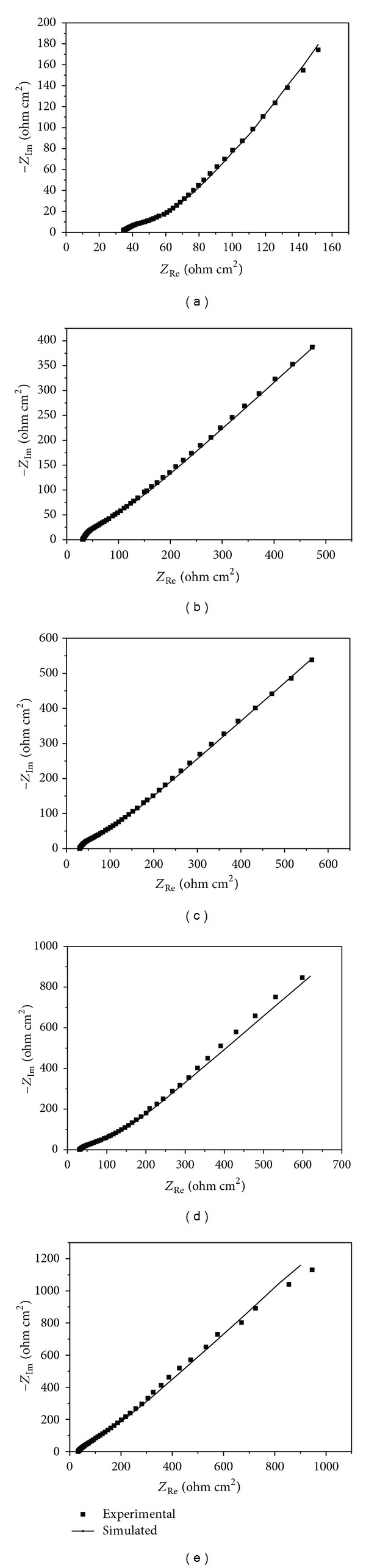
Experimental and simulated Nyquist plots of pyrite samples coated by different concentrations of TETA. (a) Uncoated; (b) coated by 1% TETA; (c) coated by 2% TETA; (d) coated by 3% TETA; (e) coated by 5% TETA.

The experimental and simulated impedance diagrams for pyrite samples coated by different concentrations of TETA are presented in Figure 7.  Table 3 shows the quantitative results for impedance which fitted using the equivalent circuit *R*_*s*_(*Q*_1_(*R*_1_ *Q*_2_)) as mentioned above. The fact of a low value of *x* ^2^, which represents the sum of quadratic deviations between experimental and calculated data suggested that the proposed circuit is suitable for explaining the EIS spectra. Because all of the experiments were carried out in the same electrolyte, the values of the solution resistance (*R*_*s*_) were almost no change.

The value of charge transfer resistance “*R*_1_” is inversely proportional to corrosion rate. The charge transfer resistance increases with the increase in concentration of TETA. *R*_1_ increased from the value of 43.7 Ω cm^2^ for the pristine pyrite to 283.6 Ω cm^2^ for the pyrite coated by highest concentration of TETA, which indicates that the corrosion of pyrite is obviously inhibited in the presence of TETA.

According to (1b), the inhibition efficiencies (IE) have been calculated. The obtained results show that the inhibition efficiency increases, while the charge transfer resistance increased when the concentration of the TETA increased. The results obtained from the EIS method were in good agreement with those obtained from the polarization measurements.

## 4. Conclusion

The feasibility of using TETA as a protecting agent to reduce the oxidation of pyrite had been studied using electrochemical technique. The results show that TETA is an efficient coating agent in preventing the oxidation of pyrite, and the inhibition efficiency was more pronounced with TETA concentration. The CV measurements reveal that TETA possessed strong capability to be used as passivation agent for AMD control. The potentiodynamic polarization curves indicate that TETA inhibited both anodic pyrite dissolution and also cathodic hydrogen evolution reactions, and it acted as mixed type inhibitor in acid solution. The values of inhibition efficiency obtained from the EIS method are in good agreement with the results of polarization measurement.

## Figures and Tables

**Table 1:** Chemical composition of the studied pyrite sample.

Compound	Mass/%
FeS_2_	93.33
SiO_2_	3.38
Al_2_O_3_	1.45
MgO	0.28
Cr_2_O_3_	0.01
P_2_O_5_	0.04
K_2_O	0.74
TiO_2_	0.03
MnO	0.03
NiO	0.18
CuO	0.18
ZnO	0.20
WO_3_	0.07
PbO	0.05
As_2_O_3_	0.04

**Table 2:** Electrochemical polarization parameters and the corresponding inhibition efficiencies for pyrite coated with different concentrations of TETA.

Concentration of TETA (%)	*E*_corr_ (mV/SCE)	β_*c*_ (decade *V* ^−1^)	*β*_*a*_ (decade *V* ^−1^)	*j*_corr_ (mA cm^−2^)	*η* (%)
0%	253	9.59	7.44	0.0426	—
1%	226	8.82	6.73	0.0247	42.08
2%	206	7.91	5.96	0.0183	57.04
3%	187	7.72	5.23	0.0131	69.24
5%	100	7.70	4.53	0.0081	80.98

**Table 3:** Parameters using the circuit *R*_*s*_(*Q*_1_(*R*_1_ *Q*_2_)) and the corresponding inhibition efficiencies for pyrite coated with different concentrations of TETA.

Concentration of TETA (%)	*R*_*s*_/Ω cm^2^	*Y*_0,1_ /10^−3^ S s^*n*^ cm^−2^	*n *	*R*_1_/Ω cm^2^	*Y*_0,2_ /10^−3^ S s^*n*^ cm^−2^	*n *	*x* ^2^/10^−4^	IE (%)
0	33.63	4.21	0.8	43.77	9.15	0.5	5.36	—
1	31.04	1.24	0.8	76.91	5.70	0.5	2.14	43.09
2	30.01	1.21	0.8	96.21	4.69	0.5	1.65	54.50
3	30.56	1.41	0.8	154.3	3.89	0.6	4.02	71.63
5	32.64	1.34	0.8	250.8	1.97	0.6	2.60	82.55
